# Diagnostic and Interventional Imaging in Various Diseases

**DOI:** 10.3390/medicina60111810

**Published:** 2024-11-04

**Authors:** Romica Cergan, Mihai Dumitru, Adrian Costache

**Affiliations:** 1 Anatomy Department, Carol Davila University of Medicine and Pharmacy, 050474 Bucharest, Romania; r.cergan@gmail.com; 2 ENT Department, Carol Davila University of Medicine and Pharmacy, 050474 Bucharest, Romania; 3 Pathology Department, Carol Davila University of Medicine and Pharmacy, 050474 Bucharest, Romania; adriancostacheeco@yahoo.com

Diagnostic and interventional imaging is a cornerstone in the management of cases in various medical and surgical domains, such as neonatology, neurology, neurosurgery, otorhinolaryngology, dentistry, gynecology and urology.

Neonatal sonography is one of the newest instruments available in the diagnostic imaging newborns. The main advantage of performing lung sonography is that this imaging modality does not expose the newborn to radiation and permits serial imaging with the dynamic visualization of the progression or resolution of the pulmonary pathology. A recent study revealed that implementing lung sonography on a large scale diminished the dose of radiation by 30% per ventilated newborn [1]. It is therefore evident that sonography is the stethoscope of the future.

One domain that relies heavily on diagnostic and interventional imaging modalities is neuroimaging. Almost all improvements in the outcome and survival of neurology and neurosurgery patients in the last decade have been due to the extended availability of interventional neuroradiology procedures. However, when facing aneurysmal subarachnoid hemorrhage (ASAH), 19.7% of patients continue to die during hospitalization despite the use of endovascular interventions [2]. Moreover, ischemic stroke cases benefit from the use of a combination of contrast CT scans and MRI scans, which requires close cooperation between the neurologist and imaging specialist [3]. These data show that further improvements in both the pre-hospital and on-arrival management of patients with ASAH or ischemic stroke are required in order to reduce mortality rates further.

The viscerocranium is the anatomical region with the highest density of landmarks per voxel. The successful management of such cases relies on diagnostic imaging. Otorhinolaryngology and head and neck surgeons require numerous imaging modalities such as CT of the mastoid bone and cone beam CT of the sinuses. There is a good-to-excellent correlation between the findings of temporal bone high-resolution CT scans (HRCT) at the level of the mastoid bone and intraoperative lesions, particularly in tegmen tympani erosion, sigmoid plate dehiscence, malleus erosion, and scutum erosion [4]. The successful management of these cases at the level of the viscerocranium requires a multidisciplinary team of ear, nose and throat (ENT) surgeons, and dentists. Endodontic treatment should be conducted with careful cone beam CT imaging in order to prevent complications in cases with radix entomolaris [5]. Faster imaging equipment and targeted imaging protocols are necessary for the long-term follow up of such cases [6].

Emergency imaging requires trained diagnostic imaging specialists to be integrated into teams with general surgeons and pathologists. Without a complete imaging protocol, there are many anatomical variants that could result in the occurrence of complications and incidents during laparoscopic surgery [7].

Another task requiring extensive diagnostic procedures is the staging of oncological diseases. MRI is increasingly considered the gold standard for the presurgical staging of soft tissue tumors. Endometrial carcinoma can be detected on T2-weighted images taken after intravenous gadolinium chelates bolus caused by variations in vascularity; these images can be compared to the myometrium in order to differentiate it from the fluid filling the endometrial cavity. These real-life data show that the contrast of these tumors was assessed as low in 30 cases (67%), intermediate in 3 cases (7%) and heterogenous in 12 cases (26%) [8]. The further development of MRI protocols should focus on diminishing interobserver variability.

Imaging is highly important during the performance of radiation therapy. Novel techniques related to cervical cancer focus on adaptative strategies that reduce the radiation field and exposure of the patient [9].

Novel interventional radiology techniques have been implemented in order to undertake the minimally invasive management of pelvic pathology. An in-depth knowledge of the variability in the periprostatic vascular plexus is necessary in order to achieve the selective embolization of the prostatic parenchyma and prevent complications [10]. These procedures should be included in all core curricula of interventional radiology training.

Recent surge of infectious diseases in the aftermath of COVID-19 pandemics increased the number of imaging tests required for serial management of pathology progression. Imaging studies are needed for long term long term follow up of HIV, hepatitis and tuberculosis cases [11].

In conclusion, diagnostic and interventional imaging is the cornerstone to completing and correcting the management of complex pathologies. Medicine is now entering a new era of technology in which artificial intelligence (AI)-powered diagnostic tools will enhance the management of workflow in already crowded radiology departments.
